# Comparative Analysis of Different Extracellular Matrices for the Maintenance of Bovine Satellite Cells

**DOI:** 10.3390/ani14233496

**Published:** 2024-12-03

**Authors:** Jae Ho Han, Si Won Jang, Ye Rim Kim, Ga Rim Na, Ji Hoon Park, Hyun Woo Choi

**Affiliations:** 1 Department of Agricultural Convergence Technology, Jeonbuk National University, Jeonju 54896, Republic of Korea; jaehozizon@jbnu.ac.kr (J.H.H.); nado0721@naver.com (G.R.N.); wlgns93222@jbnu.ac.kr (J.H.P.); 2 Department of Animal Science, Jeonbuk National University, Jeonju 54896, Republic of Korea; cochon1956@naver.com

**Keywords:** bovine satellite cells, extracellular matrix, gelatin, maintenance, proliferation, cultured meat

## Abstract

Cultured meat produced using satellite cells has emerged to address issues such as overpopulation, the ethical conundrums associated with the breeding environment, and the methane gas emissions associated with factory farming. To date, however, the challenges of maintaining satellite cells in vitro and reducing the costs of the culture media are still substantial. Gelatin, collagen, and fibronectin are commonly used extracellular matrices (ECMs) that facilitate signal integration with the cells and promote cell adhesion. In this study, we compared the proliferation, cell cycle, immunocytochemistry, and expression levels of *Pax7, Pax3*, *Myf5*, *MyoD1*, and *MyoG* genes in bovine satellite cells (BSCs) cultured on gelatin-, collagen- and fibronectin-coated dishes as part of short- and long-term cultures. We observed that BSCs cultured on gelatin-coated dishes showed higher levels of Pax7 expression than BSCs cultured on collagen- and fibronectin-coated dishes in both short- and long-term cultures, indicating that BSCs cultured on gelatin effectively maintained the satellite cell population in both the short- and long-term cultures. Our study highlights that gelatin is an effective ECM for the maintenance of BSCs and the production of cultured meat.

## Simple Summary

Our aim in this study was to investigate the appropriate extracellular matrix (ECM) for the long-term culture of bovine satellite cells (BSCs), with the goal of supplying satellite cells for cultured meat. We found that BSCs cultured on gelatin were effectively maintained by expressing high levels of *Pax7* in both short- and long-term cultures.

## 1. Introduction

Satellite cells were first studied in 1961, initially focusing on signaling pathways and their role in muscle regeneration [1,2,3,4,5]. Since then, the growing global population has heightened the demand for sustainable sources of nutrition. However, sourcing nutrition from livestock poses major challenges, including excessive deforestation and increased livestock waste due to factory farming [6]. ‘Artificial meat’ or ‘cultured meat’ has emerged as a potential solution. These meats are produced in a laboratory using satellite cells, adipose cells, and a stacking material such as a scaffolds or micro-carriers [7,8]. While promising, the cost of producing meat using culture media is currently too high for mass production, and there is no effective method for maintaining satellite cells in vitro. Therefore, a continuous supply of satellite cells remains essential [9].

The satellite cells are precursors of muscle fibers. The satellite cells are located between the muscle fiber and basal lamina, along blood vessels and the extracellular matrix (ECM), collectively known as the satellite cell ‘niche’ [10]. Within this niche, when a signal occurs, satellite cells interact with surrounding cells or the ECMs and commit to myogenesis. The myogenic-committed satellite cell then begins to differentiate into myofibers [11,12,13]. The paired box 7 (Pax7) gene is known for maintaining satellite cells and regulating myogenic differentiation. However, the expression levels of *Pax7* drop significantly as it is cultured in vitro for long periods [14,15].

A number of researchers have attempted to maintain and proliferate bovine satellite cells (BSCs) in vitro by adding growth factors and cytokines to the culture medium [16,17]. FGF (fibroblast growth factor) is essential for the maintenance of satellite cells and promoting muscle regeneration [18]. IGF-1 (Insulin-like Growth Factor 1) mediates muscle hypertrophy by enhancing satellite cell activation and proliferation [19]. Interleukin-6 (IL-6) promotes the proliferation of myogenic satellite cells during muscle regeneration [20]. Additionally, signaling pathways regulate satellite cell proliferation and maintenance. Previous studies have shown that Notch and Wnt signals amplify satellite cells’ populations in vitro [21,22,23,24,25]. MAPK (Mitogen-Activated Protein Kinase) is a known initiator of myogenesis, and in BSCs, inhibiting the p38 MAPK signaling pathway regulates differentiation and enhances the expression of the Pax7 gene [14,26,27].

Fibronectin, collagen, and laminin are ECM components that exist within the satellite cell niche. In vitro, these ECM components serve as adhesives between the cells and the culture dish. These ECM components integrate signals through integrin receptors and promote cell–cell adhesion [28,29]. Collagens are one of the ECM glycoproteins that are widely abundant in tissues [30]. They are mainly produced by fibroblasts but are also secreted in chondrocytes, muscle stem cells, and committed myoblasts [31]. It has been reported that collagen type VI maintains the self-renewal of the satellite cells by regulating the muscle satellite cell niche [32]. Fibronectin can be produced by fibroblasts, chondrocytes, myocytes, and synovial cells [33]. It regulates cell adhesion and migration through integrin receptors [34]. Gelatin is the irreversible hydrolysis product of the triple helical structure of collagen that is produced by heat and enzymatic denaturation [35]. Gelatin is low-cost, non-toxic, and has a similar structure to collagen, making it suitable for many cell culture conditions [36,37,38]. However, the characteristics of BSCs cultured on gelatin-, collagen-, and fibronectin-coated dishes over short and long periods remain unclear.

In this study, we compared cell proliferation, cell cycle dynamics, immunocytochemistry (ICC), and the expression levels of *Pax7*, *Pax3*, *Myf5* (*Myogenic Factor 5*), *MyoD1* (*myogenic differentiation*), and *MyoG* (*Myogenin*) genes in BSCs cultured on gelatin-, collagen-, and fibronectin-coated dishes in both short- and long-term cultures. We found that the population of S phase cells was significantly higher in BSCs cultured on gelatin-coated dishes compared with those on collagen- and fibronectin-coated dishes in both short- and long-term cultures. Moreover, the expression of Pax7 was also significantly higher in gelatin-coated dishes at short- and long-term cultures. Gelatin could serve as an effective ECM for maintaining BSCs for long-term culture and for the production of cultured meat.

## 2. Materials and Methods

### 2.1. BSC Isolation and Primary BSC Culture

Chuck muscle tissues were isolated from adult Korean native cattle using previously described protocols [39]. Briefly, the chuck muscle tissues were weighed to 5 g and minced for 5 min with surgical scissors. The tissues were then dissociated with Digest Solution (1 g/mL) containing DMEM/F12 (Gibco, Carlsbad, CA, USA, #11320-033), 0.25% trypsin-EDTA (TE) (Gibco, #25200-072), Collagenase 2 (Worthington, Lakewood, NJ, USA, #CLS-2, 5 g), DispaseII (Roche, Indianapolis, IN, USA, #4942078001, 1 U/mL), and 10% Antibiotic-Antimycotic (A.A., Gibco, #15240062) for 2 h at 37 °C. Each muscle fragment was neutralized with neutralized media containing DMEM (Gibco, #11885092) and 15% Fetal Bovine Serum (FBS) (Gibco, 16000-044, 26140079) supplemented with 1% A.A. The mixture was centrifuged at 80× *g* for 3 min at 4 °C. Supernatant was collected and filtered through a 100 μm strainer, followed by a 40 μm cell strainer. Red blood cell (RBC) lysing buffer (Sigma-Aldrich, St. Louis, MO, USA, #R7757-100 mL) was used to lyse the red blood cells, which were then washed with PBS twice. Cells were then reconstituted with primary culture media containing Ham’s F-10 (Gibco, #15240-062), 20% FBS, 1% A.A., basic fibroblast growth factor (bFGF) (R&D System, Minneapolis, MN, USA, #223-FB-500/CF, 5 ng/mL), and Primocin (Invivogene, Pak Shek Kok, New Territories, Hong Kong, ant-pm-2, 100 μg/mL) until a cell density of 70–80% was achieved. For the experiment, primary culture media was replaced with culture media where Primocin was discarded and A.A. was substituted with penicillin–streptomycin (PS). The muscle-derived cells (passage 0) were cultured on gelatin-coated dishes.

For the bovine satellite cells’ purification, cells were reconstituted with FACS buffer containing 1% BSA (Sigma, St. Louis, MO, USA, #A8412) with PBS and stained with APC anti-human CD29 antibody (1:20, BioLegend, San Diego, CA, USA, #303008), PE-CyTM7 anti-human CD56 antibody (1:20, BD Bioscience, Franklin Lakes, NJ, USA, #335826), FITC anti-sheep CD31 (1:10, Bio-Rad, Hercules, CA, USA, #MCA1097F), FITC anti-sheep CD45 (1:10, Bio-Rad, #MCA220F), and Hoechst 34580 (1 μg/mL, Invitrogen, Carlsbad, CA, USA, H21486) for 45 min in ice. The CD29^+^ CD56^+^ CD31^−^ CD45^−^ cell population was sorted using a BD FACS Aria III (BD bioscience, Franklin Lakes, NJ, USA). The pure bovine satellite cells from the isolated muscle-derived cells using antibodies were seeded on gelatin-coated dishes (passage 1). The experiment was conducted from passage 3 to passage 10 to identify the appropriate ECMs for the long-term maintenance of BSCs.

### 2.2. BSC Culture on ECM Coating

BSCs were cultured on dishes coated with 0.1% gelatin (Sigma-Aldrich, #G1393), 20 μg/mL of fibronectin (Sigma-Aldrich, #FC010), and 35 μg/mL of collagen (Sigma-Aldrich, #C8919). BSCs were seeded in 100 mm and 6-well plate, 4-well plate, and 96-well plate with cell quantities of 1 × 10^6^, 1.8 × 10^5^, 5 × 10^4^, and 5 × 10^3^ respectively. Media were changed every day and passaged every 3 days.

### 2.3. Cell Proliferation Analysis

BSCs were seeded in a 96-well plate coated with gelatin, collagen, and fibronectin. BSCs were seeded in every well at a density of 2 × 10^4^. Cells were then cultured in culture media for 3 days. Growth was detected using a Cell Counting Kit-8 (CCK-8, Dojindo, Kumamoto, Japan #CK04-11). Cells were treated with CCK-8 solution according to manufacturer’s instructions and incubated at 37 °C for 3 h. Proliferation was measured using a microplate reader with a wavelength of 450 nm.

### 2.4. Cell Cycle Analysis

BSCs were collected during short- (P2) and long-term (P10) culture. Cells were seeded in a 35 mm dish at a density of 18 × 10^4^. For cell cycle analysis, early and late cells were detached using 0.25% trypsin-EDTA and neutralized with neutralized media containing DMEM, 15%FBS, and 1% A.A. Cells were then washed with cold PBS (containing 1% BSA), fixed with 70% ethanol for 5 min at 4 °C, and then centrifuged 850× *g* at 4 °C for 5 min. Ethanol was removed and cells were washed with PBS twice, after which 100 μg/mL of RNase A (Sigma-Aldrich, #70856) was added, as was 25 μg/mL of Propidium iodide (PI) (Bio Legend, San Diego, CA, USA, #421301) with PBS. Cells were then analyzed by flow cytometry with a blue laser (excitation 488 nm).

### 2.5. Immunofluorescence Staining of BSCs and Analysis of Expressed Pax7 and MyoD1

BSCs were seeded in a 4-well plate at a density of 5 × 10^4^ and cultured for three days. Cells were then washed with PBS twice, fixed with 4% paraformaldehyde for 20 min at 4 °C, and then washed with PBS three times. Cells were then incubated in blocking solution (washing solution + 3% bovine serum Albumin (Bovogen, Keilor East, Australia, Bovostar) and washing solution (0.3% Triton X-100 (Gibco, 10010-023)) with PBS for 2 h. After blocking, cells were washed again with washing solution. Cells were then stained overnight with primary antibodies against anti-MyoD1 (Polyclonal, 1:200, Proteintech, Rosemont, IL, USA) and Pax7 (anti-paired box 7) (Pax7 monoclonal, 1:50, DHSB, Iowa, IA, USA) at 4 °C. Cells were then stained with Alexa488 anti-mouse (Invitrogen, USA, A11001) antibodies and Alexa568 labeled anti-rabbit (Invitrogen, USA, A11004) antibodies at room temperature for 2 h and stained with 1 μg/mL of 4′6-diamidino-2-phenylindole (DAPI) for 30 min. After DAPI staining, cells were washed with washing solution for 10 min. BSCs stained with Pax7 and MyoD1 was captured with Leica DFC 9000 (Deerfield, IL, USA) at 200× magnification. The number of expressed Pax7 and MyoD1 was calculated as a ratio of the total DAPI count.

### 2.6. Gene Expression Analysis by qRT-PCR

RNA was extracted from cells using an AccuPrep^®^ Universal RNA Extraction kit (Bioneer, Seoul, Republic of Korea) following the manufacturer’s protocol. Then, 1 μg of total RNA was reverse-transcribed with Accupower^®^ CycleScript RT Premix (Bioneer, Seoul, Republic of Korea) consistent with the manufacturer’s instructions. Relative gene expression was performed in triplicate using Powerup SYBR Green master mix (Applied Biosystem, Carlsbad, CA, USA). The primers used for qRT-PCR were as follows: GapDH sense 5′-CACCCTCAAGATTGTCAGC-3′, GapDH antisense 5′-TAAGTCCCTCCACGATGC-3′, Pax7 sense 5′-CTCC CTCT GAAG CGTA AGCA-3′, Pax7 antisense 5′-GGGT AGTG GGTC CTCT CGAA-3′, Pax3 sense 5′-CAAAGCTTACAGAGGCCCGA-3′, Pax3 antisense 5′-GGTCTCTGACAGCTGGTACG-3′, Myf5 sense 5′-TCTATCTCTCTGCTGTCCAGGC-3′, Myf5 antisense 5′-AACTCGTCCCCGAACTCAC-3′, MyoD1 sense 5′-TTTGCCAGAGCAGGAGCCCCTC-3′, MyoD1 antisense 5′-TGGACTCTTGGGCCAACTTGAGAT-3′, MyoG sense 5′-GCGCAGACTCAAGAAGGTGA-3′, and MyoG antisense 5′-TGCAGGCGCTCTATGTACTG-3′.

### 2.7. Statistical Analysis

All experiments were performed three times, and the data of all repetitions of each experiment were collated and expressed as means ± standard error (SE) of the mean. Statistical tests were conducted using SAS version 9.4 (SAS Institute Inc., Cary, NC, USA), and statistical differences were performed with Student’s *t*-test or variance (ANOVA) followed by Duncan’s Multiple Range Test for post hoc comparisons. A *p*-value < 0.05 was regarded as significant.

## 3. Results

### 3.1. Analysis of the Proliferation and Cell Cycle of BSCs Cultured on Gelatin-, Collagen- and Fibronectin-Coated Dishes in Short- and Long-Term Cultures

**Figure 1:**
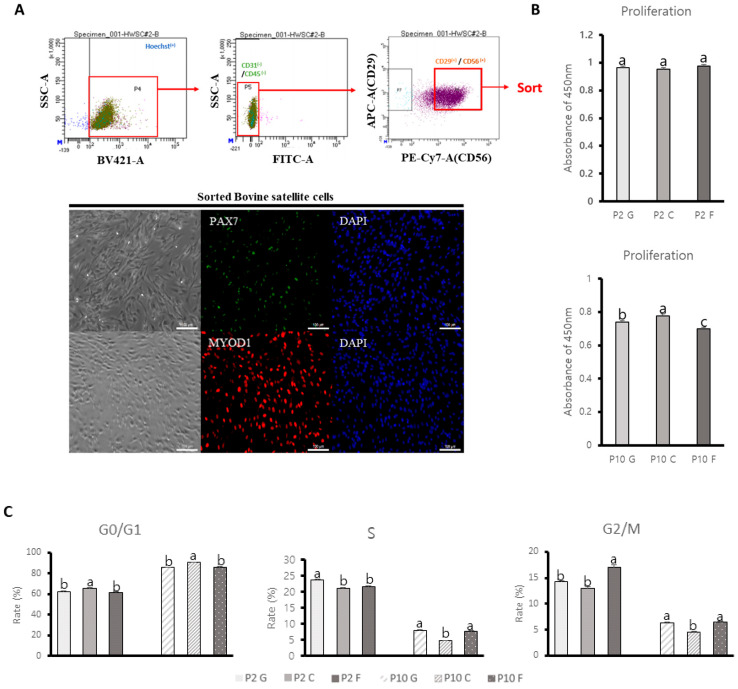
(**A**) Bovine satellite cells were sorted by CD29 (APC), CD31 (FITC), CD45 (FITC), and CD56 (PE−Cy7) antibodies and Hoechst. Cells were first distinguished as live or dead cells by Hoechst. CD31 and CD45 negative cells were then distinguished. Next, CD29 and CD56 double-positive cells were sorted. (**B**) The isolated CD29^+^, CD56^+^, CD31^−^, and CD45^−^, bovine satellite cells were stained with PAX7 (green), MYOD1 (red) antibody, and DAPI (blue). Scale bar indicates 100 μm. (**B**) Comparative analysis of cell proliferation of bovine satellite cells cultured on gelatin-, fibronectin-, and collagen-coated dishes for short (P2)- and long (P10)-term culture (*p* < 0.001). *n* = 9. Each letter (a, b, c) represents significant differences. Values are presented as means ± SE. (**C**) The cell cycles of the bovine satellite cells were stained with Propidium iodide (PI). The bovine satellite cultured on gelatin-, fibronectin-, and collagen-coated dishes for short- and long-term culture were then analyzed with flow cytometry. n = 3. Each letter (a, b, c) represents significant differences (*p* < 0.001). Values are presented as means ± SE.

**Figure 2:**
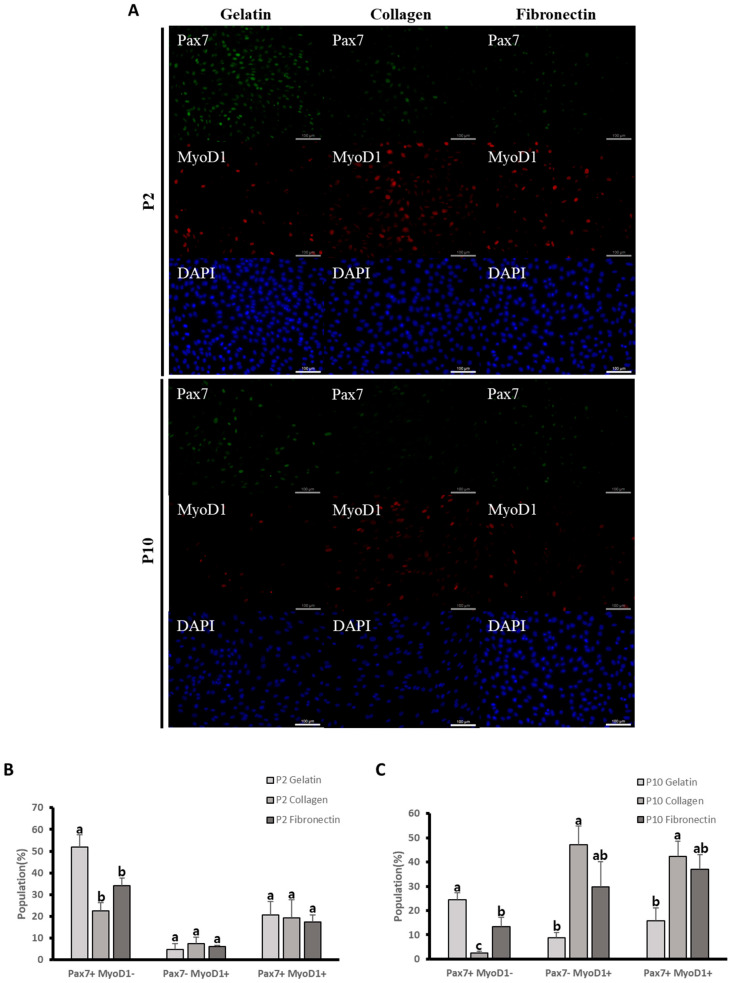
(**A**) Bovine satellite cells cultured on gelatin-, fibronectin-, and collagen-coated dishes at short- and long-term culture were stained with PAX7 (green), MYOD1 (red) antibody, and DAPI (blue). Scale bar indicates 100 μm. (**B**) Comparative analysis of Pax7^+^ MyoD1^−^, Pax7^−^ MyoD1^+^, and Pax7^+^ MyoD1^+^ cell populations between gelatin, fibronectin, and collagen in short-term culture. Population of Pax7^+^ MyoD1^−^ (*p* < 0.01) n = 3. Each letter (a, b) represents significant differences. Values are presented as means ± SE. (**C**) Comparative analysis of Pax7^+^ MyoD1^−^, Pax7^−^ MyoD1^+^, and Pax7^+^ MyoD1^+^ cell populations between gelatin, fibronectin, and collagen in long-term culture. Pax7^+^ MyoD1^−^ (*p* < 0.005), Pax7^−^ MyoD1^+^ (*p* < 0.05), Pax7^+^ MyoD1^+^ (*p* < 0.05), n = 3. Each letter (a, b, c) represents significant differences. Values are presented as means ± SE.

We first sorted the pure bovine satellite cells using the surface markers of BSCs (CD29 and CD56). Live cells were initially sorted using Hoechst staining followed by secondary sorting of the CD31 and CD45 negative population. The CD29 and CD56 positive population was sorted as pure satellite cells (Figure 1A). We next confirmed the sorted satellite cells by ICC, separating those that expressed Pax7 and MyoD1 (Figure 1A). The culture dishes were coated with 0.1% gelatin, which is commonly used in various cell types and species [40,41,42]. Fibronectin was coated at 20 μg/mL, which was the most efficient cell adhesive in MCP-5 cells [43] and had higher proliferation in porcine satellite cells [44]. The recommended concentration for collagen coating is typically 57.6–96 μg/mL in a 6-well plate. However, we found collagen coatings to be cost ineffective. We minimized the collagen concentration to 35 μg/mL in 6-well plates. To evaluate the growth of BSCs cultured on gelatin, collagen, and fibronectin coatings, we analyzed cell proliferation in both short-term (passage 2, P2) and long-term (passage 10, P10) cultures. There were no significant differences in cell proliferation among the three different ECMs at short-term culture (Figure 2A). However, the BSCs cultured on collagen-coated dishes exhibited the highest proliferation at long-term culture (Figure 2A) (*p* < 0.001). We next evaluated the cell cycle of BSCs cultured on gelatin, collagen, and fibronectin in both short- and long-term cultures. The population of G0/G1 phase cells in BSCs cultured on collagen-coated dishes was significantly higher in both short- and long-term cultures compared with gelatin- and fibronectin-coated dishes (Figure 2B) (*p* < 0.001).

The S phase population was significantly higher in BSCs cultured on gelatin-coated dishes than in those cultured on collagen- or fibronectin-coated dishes in both short-term cultures (Figure 2B) (*p* < 0.001). The population of the G2/M phase was significantly higher in BSCs cultured on fibronectin-coated dishes in both short- and long-term cultures (Figure 2B) (*p* < 0.001). Our results indicate that the BSCs being cultured on different ECMs could influence the cell proliferation and cell cycle through short- and long-term culture.

### 3.2. Comparative Analysis of Bovine Satellite Cells’ State Between Three Different ECMs

To determine the state of the BSCs cultured on the different ECMs, we first stained PAX7 and MYOD1 using ICC method (Figure 2A). We next analyzed the population of Pax7^+^ MyoD1^−^ (quiescence), Pax7^+^ MyoD1^+^ (activated satellite cells), and Pax7^−^ MyoD1^+^ (myoblasts). To determine the BSCs’ states at long-term culture depending on the different ECMs, we stained PAX7 and MYOD1 using the ICC method at long-term culture (Figure 2A). The population rate of expressed Pax7^+^ MyoD1^−^ was significantly higher in BSCs cultured on gelatin-coated dishes at short-term culture (*p* < 0.01) (Figure 2B). The population rate of expressed Pax7^+^ MyoD1^−^ was significantly higher in BSCs cultured on gelatin (*p* < 0.005) (Figure 2C). However, the population of Pax7^−^ MyoD1^+^ and Pax7^+^ MyoD1^+^ was significantly higher in BSCs cultured on collagen-coated dishes (*p* < 0.05) (Figure 2C). Our results indicate that BSCs being cultured on gelatin-coated dishes affected the population rate of BSCs that expressed only Pax7, while BSCs being cultured on collagen-coated dishes affected the population rate that co-expressed Pax7 and MyoD1, indicating that the extracellular matrix could influence the BSCs population rate.

### 3.3. Comparative Analysis of Gene Expression of BSCs Cultured on Gelatin-, Collagen-, and Fibronectin-Coated Dishes at Short- and Long-Term Cultures

**Figure 3:**
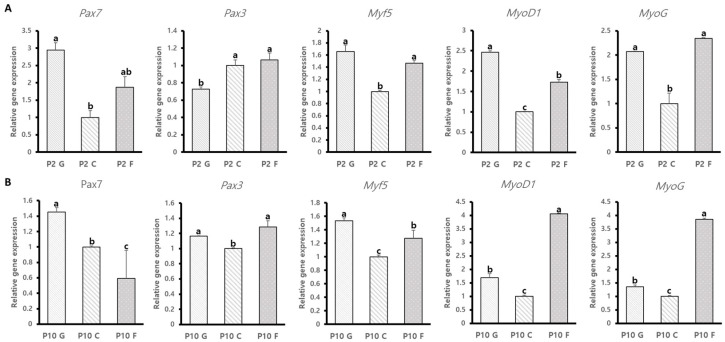
(**A**) Gene expression levels of Pax7, Pax3, Myf5, MyoD1, and MyoG in BSCs cultured on different ECMs in short-term culture (n = 3). Significant differences are indicated by different letters (a, b, c; *p* values: Pax7, Pax3, Myf5 < 0.005; MyoD1, MyoG < 0.0001). Values are presented as means ± SE. (**B**) Gene expression levels of Pax7, Pax3, Myf5, MyoD1, and MyoG in BSCs cultured on different ECMs in long-term culture (n = 3). Significant differences are indicated by different letters (a, b, c; *p* values: Pax7, Myf5 < 0.005; Pax3 < 0.05; MyoD1, MyoG < 0.0001). Values are presented as means ± SE.

It is known that the expression of Pax7 and MyoD1 in BSCs continuously decreases as they are cultured for the long term in vitro [14]. To determine the gene expression levels of BSCs cultured on different ECMs, we analyzed the expression levels of Pax7, Pax3, and myogenic regulator factors (Myf5, MyoD1, and MyoG) in BSCs cultured on gelatin-, collagen-, and fibronectin-coated dishes at short- and long-term cultures. The *Pax7* expression levels were significantly elevated in BSCs cultured on gelatin-coated dishes in both short- and long-term culture (*p* < 0.005) (Figure 3A,B). In short-term culture, *Myf5* and *MyoG* expression levels were markedly higher in BSCs cultured on both gelatin- and fibronectin-coated dishes (*p* < 0.001) (Figure 3A,B). At long-term culture, *Myf5* expression levels remained significantly higher in BSCs cultured on gelatin (*p* < 0.005), while the expression levels of *MyoG* were notably higher in BSCs cultured on fibronectin-coated dishes (*p* < 0.0001) (Figure 3A,B). The Pax3 expression levels followed a similar pattern to MyoG, with higher expression levels in BSCs cultured on collagen- and fibronectin-coated dishes compared with gelatin-coated dishes during short-term culture. However, only fibronectin-coated dishes had notably higher expression levels of *Pax3* at long-term culture (*p* < 0.05) (Figure 3A,B). The expression levels of the MyoD1 gene were significantly higher in BSCs cultured on gelatin-coated dishes at short-term culture; however, the expression levels of the MyoD1 gene were significantly higher in fibronectin-coated dishes at long-term culture (*p* < 0.001) (Figure 3A,B). Our results showed that BSCs cultured on gelatin could be maintained better than BSCs cultured on collagen- or fibronectin-coated dishes. Additionally, BSCs cultured on fibronectin-coated dishes could have a larger population of myogenic committed satellite cells compared with those cultured on gelatin- or collagen- coated dishes.

## 4. Discussion

Research has been devoted to maintaining BSCs in vitro through the regulation of growth factors, signaling pathways, and ECMs. The expression of the Pax7 gene in porcine satellite cells has been shown to be significantly higher when fibronectin-coated dishes are used compared with other ECMs (i.e., collagen, gelatin, matrigel, laminin) [29]. To date, the characteristics of BSCs cultured on gelatin, collagen, and fibronectin have remained unclear.

In the present study, we analyzed the proliferation, cell cycle, ICC, and expression levels of *Pax7*, *Pax3*, and the myogenic regulator factors *Myf5*, *MyoD1*, and *MyoG* genes of BSCs cultured on different ECMs. We found that the populations in the S phase were significantly higher in BSCs cultured on gelatin-coated dishes in both short- and long-term cultures. The population rate of Pax7^+^ MyoD1^−^ expression was significantly higher in gelatin-coated dishes compared with collagen- or fibronectin-coated dishes in both short- and long-term cultures (Figure 2B,C). Our results also confirmed that the expression levels of the *Pax7* gene were significantly higher in gelatin-coated dishes from both short- and long-term cultures. However, as BSCs were cultured for long periods, the expression levels of *MyoD1* and *MyoG* genes were ultimately significantly higher when fibronectin coated-dishes were used.

Fibronectins interact with β1-integrin and collaborate with FGF to augment the phosphorylation of ERK and the AKT signaling pathway, which regulate cell proliferation [45]. In a prior study, porcine satellite cells cultured on fibronectin expressed significantly higher levels of Pax7 compared with those cultured on gelatin [29]. Moreover, our previous results demonstrated that porcine satellite cells cultured on fibronectin showed higher proliferation and expression of Pax7 by ICC. We also previously confirmed high expression levels of *Pax7* as the concentration of fibronectin falls in porcine satellite cells [44].

Collagen is composed of three amino acids: proline, glycine, and hydroxyproline, which combine to form a triple helix structure that determines the structural form of the protein. There are 28 various types of collagen discovered in the human body, and the most common types of collagens are type I to type IV, with type I accounting for more than 90 percent [46]. Type I collagen in myoblasts substantially decreases as it differentiates, whereas the addition of collagen inhibits the differentiation of C2C12 cells [47]. A previous study revealed that impaired collagen type VI in C2C12 cells lost the ability to support self-renewal and muscle regeneration after injury in vivo. However, impaired collagen type VI C2C12 cells were able to recover the satellite cells’ population when cultured on collagen type VI-coated dishes in vitro, indicating that collagen type VI regulates the self-renewal and regeneration of mouse muscle satellite cells [32].

Gelatin is widely used in cell cultures to improve the adhesion of cells to the culture dish. Ovine satellite cells cultured on pigskin gelatin have been found to initially have fewer total cells than those cultured on bovine fibronectin-coated dishes; however, the cells cultured on gelatin had a larger number of total cells at the end of the proliferation period. Ovine satellite cells cultured on gelatin have also been reported to enhance their differentiation capability by forming large myotubes that are more branched and contain more nuclei [48]. One prior study confirmed that cod-skin gelatin hydrolysates (CGHs), which are prepared by hydrolyzing gelatin with alkaline protease and trypsin, reduced MMP-1 expression, subsequently decreasing type I collagen in fibroblasts under UV irradiation conditions. Among CGHs, two peptides have been shown to inhibit p-ERK and p-p38 in the MAPK pathway [49]. Notably, the inhibition of p-p38 by p38i in BSCs leads to an increase in the Pax7 expression levels [14].

When the expression of Pax7 is high alone in mouse muscle stem cells, it is associated with quiescent muscle stem cells, which are known for their slow entry into the cell cycle, low metabolic activity, and effective engraftment in vivo [50]. In another study, it was shown that the population of mouse satellite cells expressing Pax7^+^ MyoD1^−^ was higher on non-coated dishes compared with those on matrigel- and collagen VI-coated dishes [32].

Our results showed that BSCs cultured on gelatin exhibited a significantly higher population of cells expressing Pax7^+^ MyoD1^−^ in both short-term and long-term cultures compared with those cultured on other ECMs (Figure 2B,C). In contrast, BSCs cultured on fibronectin and collagen exhibited higher populations of cells expressing both Pax7 and MyoD1, as well as MyoD1 alone, at long-term culture (Figure 2B,C). In addition, previous studies have shown that high expression of Pax7 combined with low expression of Myf5 is associated with a quiescent and self-renewing state, while high expression of both Pax7 and Myf5 is linked to activated satellite cells, leading to myogenic differentiation [51]. Our results demonstrate that BSCs cultured on gelatin had significantly higher levels of *Pax7* and *Myf5* expression at long-term culture compared with those cultured on fibronectin and collagen (Figure 3A,B). This finding suggests that the BSCs cultured on gelatin are more effective at maintaining themselves over time compared those cultured with other ECMs. ECMs seemed to affect the population rate of BSCs in vitro, and further research is needed to determine the relationship between the population of BSCs and the ECMs. Effective maintenance and high growth rates of BSCs are essential for the production of bovine-derived cultured meat. The development of methods for maintaining and expanding BSCs in vitro is necessary for ensuring a continuous supply of BSCs.

## 5. Conclusions

In our present study, we confirmed that gelatin maintains the BSCs’ population during long-term culture. Gelatin is considered an appropriate ECM for supplying cells needed for cultured meat production due to its cost advantage compared with other ECMs.

## Data Availability

The dataset used and/or analyzed during the current study is available from the corresponding author on reasonable request.

## References

[1] Schultz E., Jaryszak D.L., Valliere C.R. (1985). Response of satellite cells to focal skeletal muscle injury. Muscle Nerve.

[2] Blaveri K., Heslop L., Yu D.S., Rosenblatt J.D., Gross J.G., Partridge T.A., Morgan J.E. (1999). Patterns of repair of dystrophic mouse muscle: Studies on isolated fibers. Dev. Dyn..

[3] Gross J.G., Morgan J.E. (1999). Muscle precursor cells injected into irradiated mdx mouse muscle persist after serial injury. Muscle Nerve.

[4] Snow M.H. (1978). An autoradiographic study of satellite cell differentiation into regenerating myotubes following transplantation of muscles in young rats. Cell Tissue Res..

[5] Mauro A. (1961). Satellite cell of skeletal muscle fibers. J. Biophys. Biochem. Cytol..

[6] Tuomisto H.L., de Mattos M.J.T. (2011). Environmental Impacts of Cultured Meat Production. Environ. Sci. Technol..

[7] Post M.J. (2012). Cultured meat from stem cells: Challenges and prospects. Meat Sci..

[8] Bonny S.P.F., Gardner G.E., Pethick D.W., Hocquettez J.F. (2015). What is artificial meat and what does it mean for the future of the meat industry?. J. Integr. Agric..

[9] Post M.J., Levenberg S., Kaplan D.L., Genovese N., Fu J.A., Bryant C.J., Negowetti N., Verzijden K., Moutsatsou P. (2020). Scientific, sustainability and regulatory challenges of cultured meat. Nat. Food.

[10] Morgan J.E., Partridge T.A. (2003). Muscle satellite cells. Int. J. Biochem. Cell Biol..

[11] Chal J., Pourquie O. (2017). Making muscle: Skeletal myogenesis in vivo and in vitro. Development.

[12] Zammit P.S. (2017). Function of the myogenic regulatory factors Myf5, MyoD, Myogenin and MRF4 in skeletal muscle, satellite cells and regenerative myogenesis. Semin. Cell Dev. Biol..

[13] Hernandez-Hernandez J.M., Garcia-Gonzalez E.G., Brun C.E., Rudnicki M.A. (2017). The myogenic regulatory factors, determinants of muscle development, cell identity and regeneration. Semin. Cell Dev. Biol..

[14] Ding S., Swennen G.N.M., Messmer T., Gagliardi M., Molin D.G.M., Li C., Zhou G., Post M.J. (2018). Maintaining bovine satellite cells stemness through p38 pathway. Sci. Rep..

[15] Ding S.J., Wang F., Liu Y., Liz S., Zhou G.H., Hu P. (2017). Characterization and isolation of highly purified porcine satellite cells. Cell Death Discov..

[16] Clegg C.H., Linkhart T.A., Olwin B.B., Hauschka S.D. (1987). Growth factor control of skeletal muscle differentiation: Commitment to terminal differentiation occurs in G1 phase and is repressed by fibroblast growth factor. J. Cell Biol..

[17] Liu Y., Schneider M.F. (2014). FGF2 activates TRPC and Ca(2+) signaling leading to satellite cell activation. Front. Physiol..

[18] Pawlikowski B., Vogler T.O., Gadek K., Olwin B.B. (2017). Regulation of skeletal muscle stem cells by fibroblast growth factors. Dev. Dyn..

[19] Florini J.R., Ewton D.Z., Coolican S.A. (1996). Growth hormone and the insulin-like growth factor system in myogenesis. Endocr. Rev..

[20] Kami K., Senba E. (1998). Localization of leukemia inhibitory factor and interleukin-6 messenger ribonucleic acids in regenerating rat skeletal muscle. Muscle Nerve.

[21] Qin L., Xu J., Wu Z., Zhang Z., Li J., Wang C., Long Q. (2013). Notch1-mediated signaling regulates proliferation of porcine satellite cells (PSCs). Cell. Signal..

[22] Bjornson C.R., Cheung T.H., Liu L., Tripathi P.V., Steeper K.M., Rando T.A. (2012). Notch signaling is necessary to maintain quiescence in adult muscle stem cells. Stem Cells.

[23] Mourikis P., Sambasivan R., Castel D., Rocheteau P., Bizzarro V., Tajbakhsh S. (2012). A critical requirement for notch signaling in maintenance of the quiescent skeletal muscle stem cell state. Stem Cells.

[24] Le Grand F., Jones A.E., Seale V., Scimè A., Rudnicki M.A. (2009). Wnt7a activates the planar cell polarity pathway to drive the symmetric expansion of satellite stem cells. Cell Stem Cell.

[25] Eliazer S., Muncie J.M., Christensen J., Sun X., D’Urso R.S., Weaver V.M., Brack A.S. (2019). Wnt4 from the niche controls the mechano-properties and quiescent state of muscle stem cells. Cell Stem Cell.

[26] Kondoh K., Sunadome K., Nishida E. (2007). Notch signaling suppresses p38 MAPK activity via induction of MKP-1 in myogenesis. J. Biol. Chem..

[27] Weston A.D., Sampaio A.V., Ridgeway A.G., Underhill T.M. (2003). Inhibition of p38 MAPK signaling promotes late stages of myogenesis. J. Cell Sci..

[28] Schlie-Wolter S., Ngezahayo A., Chichkov B.N. (2013). The selective role of ECM components on cell adhesion, morphology, proliferation and communication in vitro. Exp. Cell Res..

[29] Wilschut K.J., Haagsman H.P., Roelen B.A. (2010). Extracellular matrix components direct porcine muscle stem cell behavior. Exp. Cell Res..

[30] Theocharis A.D., Manou D., Karamanos N.K. (2019). The extracellular matrix as a multitasking player in disease. FEBS J..

[31] Loreti M., Sacco A. (2022). The jam session between muscle stem cells and the extracellular matrix in the tissue microenvironment. npj Regen. Med..

[32] Urciuolo A., Quarta M., Morbidoni V., Gattazzo F., Molon S., Grumati P., Montemurro F., Tedesco F.S., Blaauw B., Cossu G. (2013). Collagen VI regulates satellite cell self-renewal and muscle regeneration. Nat. Commun..

[33] Dalton C.J., Lemmon C.A. (2021). Fibronectin: Molecular structure, fibrillar structure and mechanochemical signaling. Cells.

[34] Bachman H., Nicosia J., Dysart M., Barker T.H. (2015). Utilizing Fibronectin Integrin-Binding Specificity to Control Cellular Responses. Adv. Wound Care.

[35] Davidenko N., Schuster C.F., Bax D.V., Farndale R.W., Hamaia S., Best S.M., Cameron R.E. (2016). Evaluation of cell binding to collagen and gelatin: A study of the effect of 2D and 3D architecture and surface chemistry. J. Mater. Sci. Mater. Med..

[36] Lai J.Y. (2010). Biocompatibility of chemically cross-linked gelatin hydrogels for ophthalmic use. J. Mater. Sci. Mater. Med..

[37] Stevens K.R., Einerson N.J., Burmania J.A., Kao W.J. (2002). In vivo biocompatibility of gelatin-based hydrogels and interpenetrating networks. J. Biomater. Sci. Polym. Ed..

[38] Yang G., Xiao Z., Long H., Ma K., Zhang J., Ren X., Zhang J. (2018). Assessment of the characteristics and biocompatibility of gelatin sponge scaffolds prepared by various crosslinking methods. Sci. Rep..

[39] Han J.H., Yu J.S., Kim D.H., Choi H.W. (2023). The characteristics of bovine satellite cells with highly scored genomic estimated breeding value. J. Anim. Reprod. Biotechnol..

[40] Daems M., Ponomarev L.C., Simoes-Faria R., Nobis M., Scheele C.L., Luttun A., Ghesquière B., Zwijsen A., Jones E.A. (2024). Smad1/5 is acetylated in the dorsal aortae of the mouse embryo before the onset of blood flow, driving early arterial gene expression. Cardiovasc. Res..

[41] López de Maturana R., Lang V., Zubiarrain A., Sousa A., Vázquez N., Gorostidi A., Águila J., López de Munain A., Rodríguez M., Sánchez-Pernaute R. (2016). Mutations in LRRK2 impair NF-κB pathway in iPSC-derived neurons. J. Neuroinflamm..

[42] Zhou Y., Liao J., Fang C., Mo C., Zhou G., Luo Y. (2018). One-step derivation of functional mesenchymal stem cells from human pluripotent stem cells. Bio-Protocol.

[43] Dastych J., Costa J., Thompson H., Metcalfe D. (1991). Mast cell adhesion to fibronectin. Immunology.

[44] Han J.H., Jang S.W., Kim Y.R., Jang H., Shim K.S., Choi H.W. (2023). The fibronectin concentration that optimally maintains porcine satellite cells. Anim. Biosci..

[45] Rozo M., Li L., Fan C.-M. (2016). Targeting β1-integrin signaling enhances regeneration in aged and dystrophic muscle in mice. Nat. Med..

[46] Wu M., Cronin K., Crane J.S. (2024). Biochemistry, Collagen Synthesis. StatPearls.

[47] Alexakis C., Partridge T., Bou-Gharios G. (2007). Implication of the satellite cell in dystrophic muscle fibrosis: A self-perpetuating mechanism of collagen overproduction. Am. J. Physiol. Cell Physiol..

[48] Dodson M.V., Mathison B.A., Mathison B.D. (1990). Effects of medium and substratum on ovine satellite cell attachment, proliferation and differentiation in vitro. Cell Differ. Dev..

[49] Lu J.H., Hou H., Fan Y., Yang T.T., Li B.F. (2017). Identification of MMP-1 inhibitory peptides from cod skin gelatin hydrolysates and the inhibition mechanism by MAPK signaling pathway. J. Funct. Foods.

[50] Rocheteau P., Gayraud-Morel B., Siegl-Cachedenier I., Blasco M.A., Tajbakhsh S. (2012). A subpopulation of adult skeletal muscle stem cells retains all template DNA strands after cell division. Cell.

[51] Ancel S., Michaud J., Sizzano F., Tauzin L., Oliveira M., Migliavacca E., Schuler S.C., Raja S., Dammone G., Karaz S. (2024). A dual-color PAX7 and MYF5 in vivo reporter to investigate muscle stem cell heterogeneity in regeneration and aging. Stem Cell Rep..

